# Relativistic Correction from the Four-Body Nonadiabatic Exponential Wave Function

**DOI:** 10.1021/acs.jctc.4c00861

**Published:** 2024-09-27

**Authors:** Krzysztof Pachucki, Jacek Komasa

**Affiliations:** † Faculty of Chemistry, Adam Mickiewicz University, Uniwersytetu Poznańskiego 8, 61-614 Poznań, Poland; ‡ Faculty of Physics, University of Warsaw, Pasteura 5, 02-093 Warsaw, Poland

## Abstract

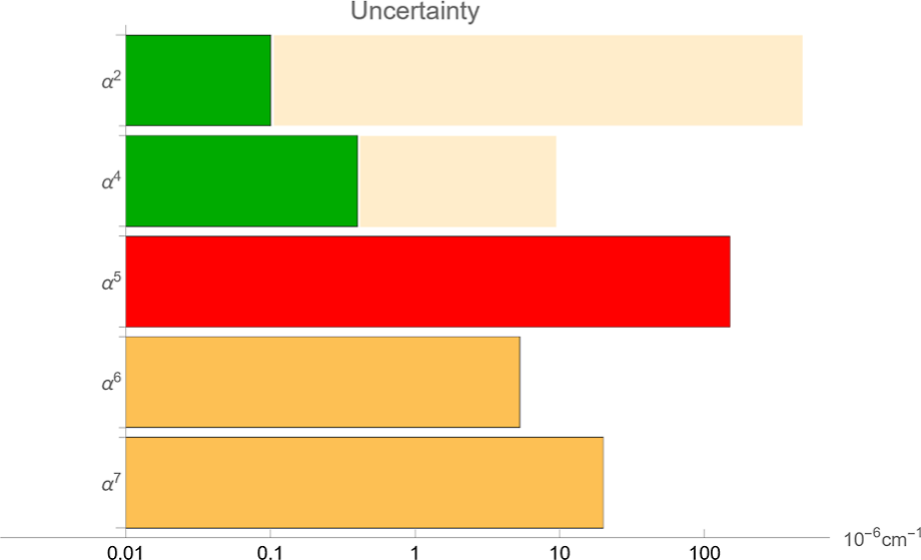

We present a method for calculating the relativistic correction in hydrogen molecules that significantly exceeds the accuracy of all the previous literature results. This method utilizes the explicitly correlated nonadiabatic exponential wave function, and thus treats electrons and nuclei equivalently. The proposed method can be applied to any rovibrational state, including highly excited ones. The numerical precision of the relativistic correction reaches several kHz (∼10^–7^ cm^–1^), which is below the best experimental accuracy.

## 1 Introduction

Since the dawn of quantum mechanics, the measurements of the hydrogen molecule spectra have been used to verify the computational methods. Modern spectroscopic experiments determine the dissociation energy of the H_2_ molecule with an accuracy of 10^–5^ to 10^–4^ cm^–1^.^1−3^ Measurements of transitions between rovibrational levels reach even higher accuracy, of the order of 10^–7^ to 10^–6^ cm^–1^.^4−10^ Achieving similar accuracy in theoretical calculations is a severe challenge. Currently, it is possible to construct a nonrelativistic wave function that fully accounts for electronic correlation and the coupling of the motion of nuclei and electrons,^11^ allowing for an accuracy better than 10^–7^ cm^–1^ for nonrelativistic energy.^12−15^ This study aims to devise a method for achieving similar accuracy for the relativistic correction.

The significance of relativistic effects in the dissociation energy *D*_0,0_ of the hydrogen molecule has been recognized long ago. In 1959, Ladik^16^ approached, although not very successfully, this issue and estimated the relativistic correction as ≈−30 cm^–1^. Fröman,^17^ using a simplistic model binding the relativistic correction with the nonrelativistic energy, arrived at +7 cm^–1^ as an estimated relativistic correction. Kołos and Wolniewicz assessed this correction as being smaller than −1.4 cm^–1^.^18,19^ The dispersion of these results, found without the use of any computer, reveals the theoretical challenges faced by the pioneering researchers in this field. Only in 1964, Kołos and Wolniewicz conducted computer-assisted calculations of the relativistic correction to *D*_0,0_ in H_2_. They obtained the first reliable estimate of this value as −0.5 cm^–1^,^20,21^ in disagreement with their previous assessment. After a 30 year hiatus, Wolniewicz revisited this topic and, using more accurate wave functions and new algorithms for calculating integrals, he obtained a value of −0.533 0 cm^–1^, which confirmed the previous estimate.^22^ Advances in computing power, wave function optimization, and techniques for accelerating the convergence of relativistic expectation values^23^ have enabled systematic improvements in the accuracy of relativistic corrections. Over time, the contribution of the finite mass of the nuclei has also been taken into account, either perturbatively^24^ or variationally.^25−27^

In our previous paper,^28^ we introduced formerly unavailable classes of integrals and showed their completeness for evaluating relativistic correction. Here, using these integrals, we develop methods for calculating the expectation values of operators included in the Breit-Pauli Hamiltonian. We illustrate the correctness and efficiency of this method by numerical results for the lowest rotational levels of H_2_ using the nonadiabatic James-Coolidge basis (naJC). It is worth emphasizing, however, that the presented method has broader application and can also be employed in other four-particle systems, such as HeH^+^, and in vibrationally and electronically excited states. Moreover, the generalization of expectation value identities and reduction of angular variables are also suitable for other nonadiabatic basis sets, e.g., nonadiabatic explicitly correlated Gaussian functions (naECG).

## 2 Relativistic Correction

Let us now introduce a formal theory of molecular levels. The total energy of a rovibrational level of a light molecule with vibrational (*v*) and rotational (*J*) quantum numbers can be expressed as a series in powers of the fine structure constant α
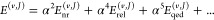
1where expansion coefficients may involve powers of ln α. The coefficients are commonly interpreted as, respectively, the nonrelativistic, relativistic, quantum electrodynamic, etc., components of the energy and can be determined in the framework of the quantum electrodynamic (QED) theory. Numerical values of these terms for specific rovibrational levels and isotopologues are available in literature.^27,29,30^

The primary objective of this work is to accurately calculate *E*_rel_^(*v*,*J*)^—the relativistic correction for rovibrational states of H_2_ and its isotopologues. This correction is given by the expectation value of the mass-dependent Breit-Pauli Hamiltonian
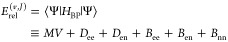
2
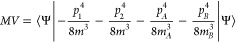
3
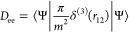
4
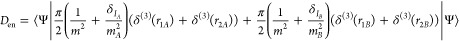
5
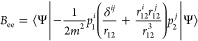
6
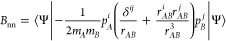
7
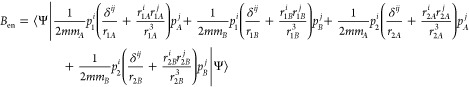
8with the nonrelativistic wave function Ψ. This wave function is obtained by solving the Schrödinger equation with the nonrelativistic Hamiltonian *H*
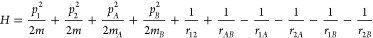
9

In the above equations, the subscripts *A* and *B* that appear alongside the symbols of mass (*m*), momentum (*p*), and coordinate (*r*) are associated with the nuclei, while the subscripts 1 and 2 are associated with the electrons. The momentum operators  are defined in the Cartesian laboratory frame. The nuclear-spin factor δ_*I*_, present in *D*_en_, depends on the nucleus’ spin *I*: δ_*I*_ equals 1 when *I* = 1/2, and 0 otherwise. It comes from the so-called Darwin term for the spin 1/2 particles. In ⟨Ψ|*H*_BP_|Ψ⟩ we have omitted all the electron spin-dependent terms because they vanish for the ground electronic state of ^1^Σ_*g*_^+^ symmetry. We have also omitted the nuclear-spin-dependent terms because we do not consider the fine and hyperfine structure in the present paper. Lastly, the term proportional to the nucleus–nucleus Dirac delta, involving strong interactions, is left out due to its negligible value.

## 3 Wave Function

We utilize the direct nonadiabatic (DNA) approach,^11^ with the wave function Ψ expanded in the basis of the nonadiabatic James-Coolidge (naJC) functions that are introduced in this section. Formally, the nonadiabatic wave function Ψ^*J*^ of a rotational level *J* contains terms depending also on the quantum number *M*, which represents the projection of the total angular momentum *J⃗* on the axis *Z* of the laboratory frame. We shall use expectation values averaged over *M*
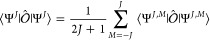
10which is equivalent to neglecting the fine and hyperfine structure. When the rotational angular momentum of nuclei couples to the electronic angular momentum *L⃗*, it forms the total angular momentum *J⃗* of the molecule. Therefore, the wave function should reflect this coupling by involving components relevant to appropriate electronic states. To distinguish between such states, we use the quantum number Λ, which is the eigenvalue of  (*n⃗* is defined below eq 15), and the inversion symmetry symbol *g* or *u* (for gerade or ungerade). These states include Σ_*g*,*u*_, Π_*g*,*u*_, Δ_*g*,*u*_, etc., and they correspond to |Λ| = 0, 1, 2, ..., respectively. The general wave function is thus represented as a sum of components with increasing Λ
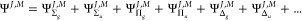
11

In the evaluation of expectation values of *H*_BP_, we limit the expansion 11 to Σ and Π terms. It is fully justified because each Λ-component of the wave function enters with a power of electron-to-reduced-nuclear-mass ratio, , thus we neglect terms with factors of , which are much smaller than our target numerical uncertainty for the relativistic correction.

The functions Ψ_Λ_^*J*,*M*^ are represented as linear expansions
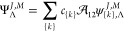
12in the following four-particle nonadiabatic James-Coolidge (naJC) basis functions
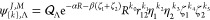
13with ζ_1_ = *r*_1*A*_ + *r*_1*B*_, η_1_ = *r*_1*A*_ – *r*_1*B*_, ζ_2_ = *r*_2*A*_ + *r*_2*B*_, η_2_ = *r*_2*A*_ – *r*_2*B*_, and . α and β denote nonlinear variational parameters, and *k*_*i*_ are non-negative integers collectively denoted as {*k*}. The inversion symmetry of the state is encoded in the sum of the two exponents *k*_2_ + *k*_3_. The basis function is symmetric with respect to the inversion when this sum is even and antisymmetric when it is odd. The preexponential factor  depends explicitly on the quantum numbers *J* and *M*, and determines the electronic angular momentum to which the basis function pertains
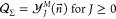
14
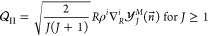
15where  or , ρ_*a*_^*i*^ = (δ^*ij*^ – *n*^*i*^*n*^*j*^)*r*_*aA*_^*j*^, and *n*^*i*^ ≡ *R*^*i*^/*R*, and where the Einstein summation convention was assumed. The solid harmonic , which carries the angular part of the nuclear variables, is related to the spherical harmonic by . The remaining, “electronic” part of the basis function will be denoted ϕ_{*k*}_, so that in short
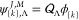
16

In eq 12, the antisymmetry projector , where the symbol *P*_12_ denotes the electron permutation operator and the internal sign is adapted to the electronic spin of the state. Finally, the linear coefficients *c*_{*k*}_ are determined variationally by solving the eigenvalue problem in the matrix form.

Details on the wave function properties, on evaluation of the nonrelativistic matrix elements, and on solving the general symmetric eigenvalue problem, were described in refs (11–14) and will not be repeated here.

## 4 Matrix Elements

In the following sections, we are going to discuss evaluation of the matrix elements
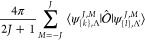
17of subsequent operators  that compose the Breit-Pauli Hamiltonian. To reduce the computational cost, we consider such an operator , which commutes with . The factor 4π in eq 17 comes from an implicit integration over angles of *R*, which from now on is pulled out in front of all matrix elements.

### 4.1 Reduction of the Angular Dependence

Summation over *M* makes use of an addition theorem for spherical harmonics, which in our notation reads
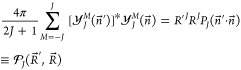
18where *P*_*J*_ is the Legendre polynomial expressible in terms of the hypergeometric function _2_*F*_1_
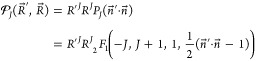
19suitable for symbolic differentiation at . Such a differentiation, , yields factors that explicitly depend on *J*. A list of such factors is placed in Supporting Information.

For example, for an operator , which contains no nuclear derivative, the reduction is as follows
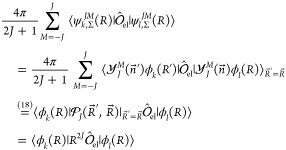
20For −∇_*R*_^2^ we get
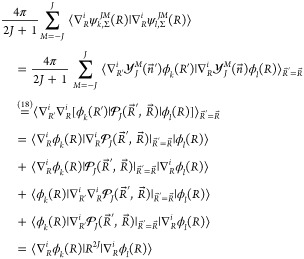
21and similarly for a Π state
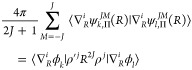
22

The elementary matrix elements  were evaluated using symbolic algebra software Wolfram Mathematica^31^ and coded in Fortran 95. In general, such matrix elements may contain thousands of terms, which prevents its explicit presentation. The simplest example of such a matrix element is the overlap integral
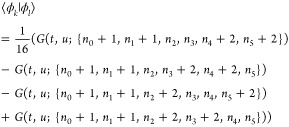
23expressed by the basic integrals *G* defined below.

### 4.2 Integrals in the Nonadiabatic James-Coolidge Basis

Matrix elements of the nonrelativistic Hamiltonian in the naJC basis are expressible by integrals of the form
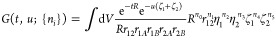
24Techniques developed by one of the authors to find such integrals were described in refs (32 and 33). However, calculating the matrix elements of the Breit-Pauli Hamiltonian requires a more extensive set of integrals, including those with a square of a variable in the denominator
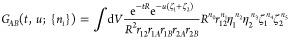
25
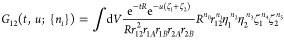
26
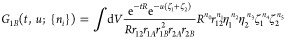
27

The remaining integrals (*G*_1*A*_, *G*_2*A*_, and *G*_2*B*_) can be obtained by permuting variables. A method for determining all such extended integrals has recently been invented,^28^ enabling the evaluation of the relativistic correction within the framework of the DNA approach using the naJC wave function.

In the following sections we shall describe methods of regularization of the relativistic operators contained in *H*_BP_: the mass velocity, the Dirac delta, and the orbit–orbit (Breit) interaction.

## 5 Mass Velocity, *MV*

The first expectation value we are going to consider consists of four *p*^4^ operators, each pertinent to one particle, see eq 3. First, we will subject it to a transformation that aims to avoid intractable integrals and speed up its numerical convergence with the increasing basis set size. For this purpose, we will add to the formula 3 a null term of the form
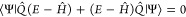
28

In the above equation, *Ĥ* is the nonrelativistic Hamiltonian (7) with the eigenvalue *E* and the eigenfunction Ψ, while Q̂ is an arbitrary operator. Assuming
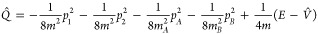
29we eliminate all *p*^4^ terms and the slowly convergent  terms. As a result, the expectation value (3) can be expressed as a sum of five terms
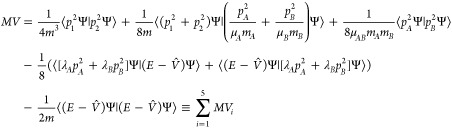
30where the following mass factors were used: , , and . All these expectation values can be calculated using the extended set of integrals mentioned in Section 4.2. Because their matrix elements are calculated with basis functions ψ_*k*,Λ_^*J*,*M*^ of both Λ = Σ and Λ = Π, three kinds of such matrix elements have to be considered: Σ–Σ, Π–Π, and Π–Σ. For each kind, the reduction of the angular variables yields a different combination of elementary matrix elements in the ϕ_*k*_ basis. Examples of such matrix elements are presented in Supporting Information.

The numerical convergence of the five terms composing the regularized formula 30 is presented in Table 1. As can be inferred from this table, at least eight significant figures can be achieved for these components, and the extrapolated total *MV* is converged up to nine significant figures. The absolute accuracy of this expectation value is limited by the uncertainty of the ⟨*p*_1_^2^Ψ|*p*_2_^2^Ψ⟩ term.

**Table 1:** Convergence of the Five Terms of Eq 30 Composing the Expectation Value *MV*, Eq 3^a^

Ω	*K*	*MV*_1_	*MV*_2_ × 10^3^	*MV*_3_ × 10^8^	*MV*_4_ × 10^3^	*MV*_5_	*MV*
11	61,152	0.32499186826	3.843967596	1.08255203165	3.880833262	–1.95862877353	–1.62591209358
12	85,904	0.32499189548	3.843967558	1.08255203121	3.880833241	–1.95862876762	–1.62591206051
13	117,936	0.32499190585	3.843967592	1.08255203106	3.880833259	–1.95862876761	–1.62591205009
14	159,120	0.32499191053	3.843967568	1.08255203189	3.880833246	–1.95862876649	–1.62591204433
	*∞*	0.324991916(5)	3.8439676(1)	1.082552032(1)	3.88083325(3)	–1.958628767(1)	–1.625912039(5)
Rel. uncert.		2 × 10^–8^	3 × 10^–8^	9 × 10^–10^	8 × 10^–9^	5 × 10^–10^	3 × 10^–9^

a Calculations performed (in a.u.) using the nonadiabatic James-Coolidge (naJC) wave function for the ground rovibrational level of H_2_ with the proton-to-electron mass ratio 1836.152673426(32) .^34^*K* is the size of the naJC basis set employed, governed by Ω—the largest shell enabled.

## 6 Dirac Delta, *D*_αβ_

The expectation value of the Dirac delta operator for two particles α and β, see eqs 4 and 5, can be evaluated directly as a numerical value of the probability density |Ψ|^2^ at *r*_αβ_ = 0. However, experience shows that such calculations have relatively poor convergence. A significantly higher rate of convergence can be achieved when the expectation value is evaluated using its regularized form^23,35^ which, for particles of the reduced mass , reads
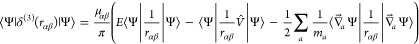
31

The right-hand-side integrals have to be evaluated in a similar manner as in the previous sections. For each, out of three kinds of matrix elements, the angular variables must be integrated out using the formulas given in Supporting Information, and then matrix elements in the elementary basis ϕ_*k*_ can be derived using symbolic algebra software.^31^ At the expense of the evaluation of three new expectation values on the right-hand side of eq 31, we can obtain results orders of magnitude more accurate than in direct calculation of ⟨Ψ|δ^(3)^(*r*_αβ_)|Ψ⟩. This is exemplified in Table 2, in which the convergence of both the direct and the regularized methods, applied to the interelectron Dirac delta expectation value, *D*_ee_, is compared. It can be concluded in this case that the regularization enables 3 orders of magnitude higher accuracy to be achieved. The convergence of the regularized expectation value of the nuclear-mass-dependent electron–nucleus Dirac delta *D*_en_ enables 10 significant figures in the extrapolated value, as shown in Table 3.

**Table 2:** Comparison of the Convergence of *D*_ee_ Calculated (in a.u.) Using the Direct and Regularized [eq 31] Methods^a^

Ω	*K*	direct	regularized
10	42,588	0.050707696496	0.050707579154
11	61,152	0.050707626769	0.050707579440
12	85,904	0.050707599520	0.050707579525
13	117,936	0.050707589148	0.050707579548
14	159,120	0.050707584473	0.050707579551
	*∞*	0.050707579(5)	0.050707579554(3)
Rel. uncert.		1 × 10^–7^	6 × 10^–11^

a The naJC wave function Ψ of the ground rovibrational level of H_2_ was used. *K* is the size of the naJC basis set employed, and Ω is the largest shell enabled.

**Table 3:** Convergence of the Operators Composing the Expectation Value of the Breit-Pauli Hamiltonian Calculated (in a.u.) Using the Nonadiabatic James-Coolidge (naJC) Wave Function for the Ground Rovibrational Level of H_2_^a^

Ω	*K*	*D*_en_	*B*_ee_	*B*_en_	*B*_nn_ × 10^6^	*E*_rel_
11	61,152	1.418309983439	–0.0462963000646	–0.00135846883424	1.83260000736	–0.20454746700
12	85,904	1.418309982287	–0.0462962993539	–0.00135846882822	1.83260000727	–0.20454743428
13	117,936	1.418309982844	–0.0462962991243	–0.00135846882836	1.83260000725	–0.20454742305
14	159,120	1.418309982463	–0.0462962990471	–0.00135846882719	1.83260000729	–0.20454741759
	*∞*	1.4183099825(5)	–0.04629629898(7)	–0.001358468828(2)	1.8326000073(1)	–0.204547412(5)
Rel. uncert.		4 × 10^–10^	2 × 10^–9^	2 × 10^–9^	6 × 10^–11^	2 × 10^–8^

a *K* is the size of the naJC basis set employed, governed by Ω—the largest shell enabled. Calculations were performed using the proton-to-electron mass ratio 1836.152673426(32).^34^

## 7 Breit Interaction, *B*_αβ_

The expectation value of the Breit interaction between two particles, α and β, cannot be directly evaluated from the formulas 6–8, as they require access to integrals beyond those discussed in Section 4.2. To address this issue, we have rearranged the original formulas to equivalent forms
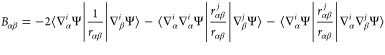
32that are suitable for the available set of integrals. The expression 32 was applied to the electron–electron, *B*_ee_, electron–nucleus, *B*_en_, and the nucleus–nucleus *B*_nn_ interactions. After necessary reduction of the angular variables it was represented in the form of elementary expectation values in the ϕ_*k*_ basis. The obtained numerical convergence with the increasing size of the basis set is shown in Table 3, from which it is evident that nine significant figures can be achieved.

## 8 Numerical Results

The convergence of the final relativistic correction for the ground energy level of H_2_ is shown in the last column of Table 3. It is apparent that it is only the ninth significant digit in the extrapolated value that is burdened with an uncertainty. We have performed analogous convergence analysis for HD and D_2_ isotopologues, obtaining (in a.u.)
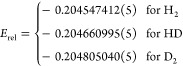
33

**Figure 1:**
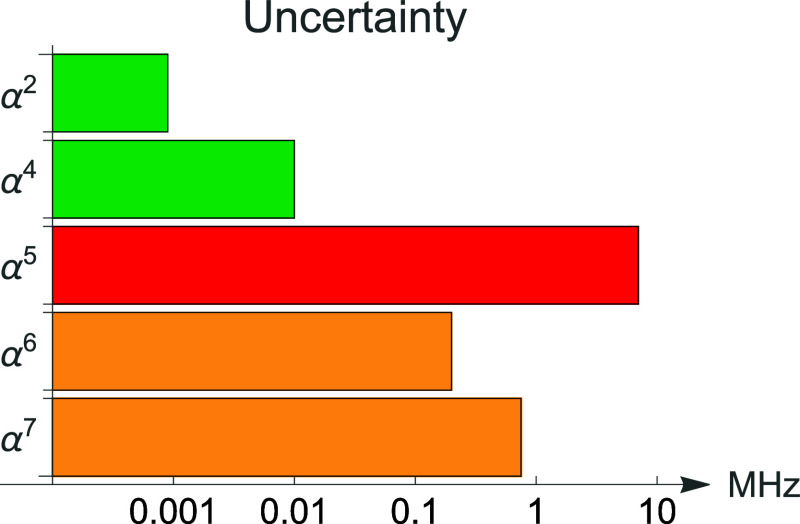
Error budget of the dissociation energy for the low-lying rotational levels of H_2_. The α^2^ and α^4^ contributions come from DNA calculation, while the remaining ones are from NAPT. Note the logarithmic scale.

The relativistic contribution to the dissociation energy can be obtained by subtracting the relativistic correction of a given rovibrational level *E*_rel_^*v*,*J*^ from the sum of the atomic relativistic corrections^36^ given by
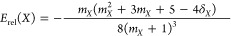
34for the nuclear mass *m*_*X*_ of the atom *X* and with δ_*H*/*T*_ = 1 and δ_*D*_ = 0. In terms of the dissociation energy of the rovibrational levels *D*_*v*,*J*_^rel^, the achieved accuracy is of the order of 10^–7^ to 10^–6^ cm^–1^ ≈ 0.003–0.03 MHz, which makes the contribution from the relativistic correction α^4^*E*_rel_^*v*,*J*^ to the overall error budget of eq 1 negligible. This result follows a similar achievement reported for the nonrelativistic component α^2^*E*_nr_^*v*,*J*^,^12−15^ so that from now on the quantum electrodynamic terms  limit the overall accuracy of the theoretical predictions. For low-lying rotational levels of H_2_ such a contribution to the total uncertainty is of the order of 2 × 10^–4^ cm^–1^ ≈ 7 MHz. The only exception is the ground rovibrational level, where the leading QED correction was calculated using the DNA method^30,37^ with the accuracy of 10^–7^ cm^–1^ ≈ 0.003 MHz. The α^6^ and α^7^ terms of the α-expansion (1) contribute the uncertainty of about 7 × 10^–6^ cm^–1^ ≈ 0.2 MHz and 3 × 10^–5^ cm^–1^ ≈ 0.8 MHz, respectively. Figure 1 provides a comprehensive view of the error budget’s components, enabling assessment of their importance.

### 8.1 Comparison with NAPT

**Figure 2:**
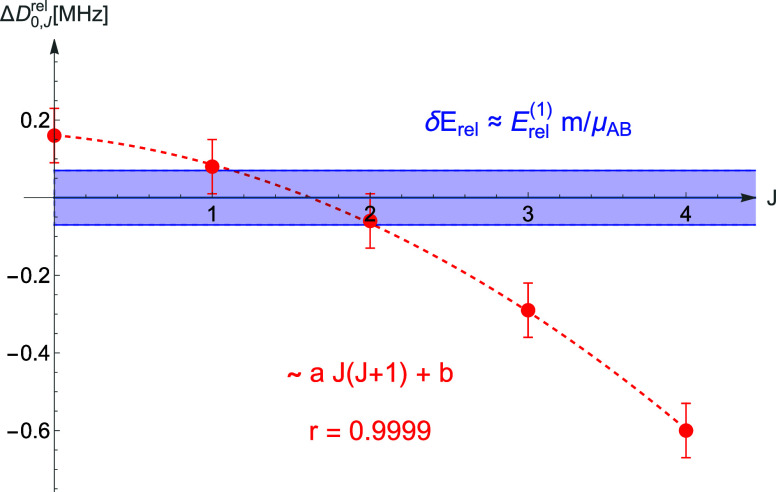
Plot of the difference between the dissociation energy of a level (0, *J*) obtained from DNA and NAPT calculations, Δ*D*_0,*J*_^rel^ (red dots), against the NAPT uncertainty (blue band) for several of the lowest rotational levels of H_2_. The Δ*D*_0,*J*_^rel^ values fit to the *a J*(*J* + 1) + *b* model with the correlation coefficient *r* = 0.9999.

Previous estimates of the relativistic correction for all the isotopologues of H_2_ were obtained in the nonadiabatic perturbation theory (NAPT) framework,^29,38,39^ which relies on the Born–Oppenheimer (BO) approximation. Within that approach, *E*_rel_ was represented as an expansion in powers of the *m*/μ_*AB*_ mass ratio around the BO value *E*_rel_^(0)^
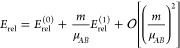
35Till now, the higher-order terms remain unknown and are the source of the uncertainty of this correction. The missing contribution was estimated using simple scaling of the first-order term by the mass factor *m*/μ_*AB*_,^40^ i.e. . So far, there have been no means to check the reliability of such an assessment. The DNA results reported in this work correspond to the expansion 35 summed up to infinity and provide a reliable tool to verify previous estimations of both the correction itself and its uncertainty. The numerical values of *E*_rel_ obtained from DNA and NAPT methods for several of the lowest rotational levels of H_2_ are compared in Table 4. In the fourth column of the table and in Figure 2, the DNA – NAPT differences are confronted with the uncertainties assigned within NAPT, while the last column of the table reveals the factor by which the uncertainty was underestimated. On the basis of these results, we can conclude that for the individual energy levels the simple scaling of the leading known term by the mass ratio does not account for the *J*-dependence of the missing terms and provides only a crude estimation. A very similar picture was found for HD and D_2_.

**Table 4:** Comparison of the Relativistic Correction to Dissociation Energy *D*_0,*J*_^rel^ (in MHz) Obtained From Direct Nonadiabatic (DNA) and Nonadiabatic Perturbation Theory (NAPT) Methods for the Lowest Rotational Levels of H_2_^a^

*J*	DNA	NAPT	difference	diff./σ_NAPT_
0	–15925.493(2)	–15925.34(7)	0.16(7)	2.3
1	–16002.87(1)	–16002.79(7)	0.08(7)	1.2
2	–16155.90(1)	–16155.96(7)	–0.06(7)	–0.9
3	–16381.19(1)	–16381.48(7)	–0.29(7)	–4.2
4	–16673.89(1)	–16674.49(7)	–0.60(7)	–8.6

a Details of the convergence of the intermediate expectation values for these levels are included in the Supporting Information. The values for *J* = 0, 1, and 2 were obtained previously.^28^

## 9 Conclusions

Recently developed integral formulas^28^ have been used to calculate expectation values of the relativistic operators included in the Breit-Pauli Hamiltonian. Both the Hamiltonian and the wave function account fully for the finite nuclear masses, significantly reducing the uncertainty of the relativistic correction. The achieved numerical convergence of the individual operators enabled stabilization of at least nine significant digits, resulting in the final accuracy of the relativistic correction of the order of 10^–9^a.u. or 10^–8^ relative. The DNA/naJC method, applied to rovibrational levels of molecular hydrogen isotopologues, yields the relativistic correction to dissociation energy with the absolute accuracy of the order of 10^–7^ to 10^–6^ cm^–1^ ≈ 0.003–0.03 MHz. Most importantly, the uncertainty of the relativistic and nonrelativistic contributions has been practically eliminated from the error budget of total energy. Therefore, future efforts to improve the accuracy of theoretical predictions will focus on the QED and higher-order corrections.

## Supporting Information Available

1. Reduction of the rotational factors. 2. Examples of Σ – Σ, Π – Π, and Π – Σ matrix elements. 3. Table S1. Convergence of the intermediate expectation values composing the relativistic correction, eq 2, for rotational levels *J* = 1–4 of H_2_ (PDF)
