# Supplementary material for: Relativistic Correction from the Four-Body Nonadiabatic Exponential Wave Function
Source: J Chem Theory Comput. 2024 Sep 27;20(19):8644–51. doi: 10.1021/acs.jctc.4c00861 (PMC11465460; doi:10.1021/acs.jctc.4c00861)
Supplement: Supplementary file 1 — ct4c00861_si_001.pdf [file ct4c00861_si_001.pdf]

Supporting Information

to

Relativistic correction from the four-body nonadiabatic exponential wave function

Krzysztof Pachucki and Jacek Komasa*

E-mail: komasa@man.poznan.pl

Contents

1 Reduction of the rotational factors 2

2 Examples of $\Sigma - \Sigma$, $\Pi - \Pi$, and $\Pi - \Sigma$ matrix elements 3

3 Table S1. Convergence of the intermediate expectation values composing the relativistic correction, Eq. (2), for rotational levels $J = 1 - 4$ of H_2 4

1 Reduction of the rotational factors

$$\mathcal{P}_J(\vec{n}'\cdot\vec{n})\Big|_{R'=R} = R^{2J} \tag{1}$$

$$\nabla^i_{R'}\,\mathcal{P}_J(\vec{n}'\cdot\vec{n})\Big|_{R'=R} = JR^{2J-2}R^m \tag{2}$$

$$\nabla^i_{R'}\nabla^n_R\,\mathcal{P}_J(\vec{n}'\cdot\vec{n})\Big|_{R'=R} = \frac{1}{2}J(J+1)R^{2J-2}\delta^{mn} + \frac{1}{2}J(J-1)R^{2J-4}R^mR^n \tag{3}$$

$$\nabla^i_R\nabla^n_R\,\mathcal{P}_J(\vec{n}'\cdot\vec{n})\Big|_{R'=R} = -\frac{1}{2}J(J-1)R^{2J-2}\delta^{mn} + \frac{3}{2}J(J-1)R^{2J-4}R^mR^n \tag{4}$$

$$\nabla^i_{R'}\nabla^i_R\,\mathcal{P}_J(\vec{n}'\cdot\vec{n})\Big|_{R'=R} = J(2J+1)R^{2J-2} \tag{5}$$

$$\nabla^i_R\nabla^i_R\,\mathcal{P}_J(\vec{n}'\cdot\vec{n})\Big|_{R'=R} = 0 \tag{6}$$

$$\nabla^i_{R'}\nabla^n_R\nabla^n_R\,\mathcal{P}_J(\vec{n}'\cdot\vec{n})\Big|_{R'=R} = 0 \tag{7}$$

$$\nabla^i_{R'}\nabla^n_R\nabla^i_R\,\mathcal{P}_J(\vec{R}',\vec{R})\Big|_{R'=R} = (J-1)J(2J+1)R^{2J-4}R^n \tag{8}$$

$$\nabla^i_{R'}\nabla^n_R\nabla^i_R\nabla^i_R\,\mathcal{P}_J(\vec{n}'\cdot\vec{n})\Big|_{R'=R} = 0 \tag{9}$$

$$\nabla^n_{R'}\nabla^n_{R'}\nabla^i_R\nabla^i_R\,\mathcal{P}_J(\vec{R}',\vec{R})\Big|_{R'=R} = 0 \tag{10}$$

$$\nabla^n_{R'}\nabla^i_{R'}\nabla^n_R\nabla^i_R\,\mathcal{P}_J(\vec{R}',\vec{R})\Big|_{R'=R} = (J-1)J(2J-1)(2J+1)R^{2J-4} \tag{11}$$

2 Examples of $\Sigma - \Sigma$, $\Pi - \Pi$, and $\Pi - \Sigma$ matrix elements

$$
\begin{aligned}
&\frac{4\,\pi}{2\,J+1}\sum_{M=-J}^{J}\left\langle p_A^2\psi_{k,\Sigma}^{J,M}\middle|p_B^2\psi_{l,\Sigma}^{J,M}\right\rangle \qquad (12)\\
&= \left\langle \nabla_A^i\nabla_A^i\phi_k \left|R^{2J}\right| \nabla_B^j\nabla_B^j\phi_l\right\rangle\\
&\quad + 2J\Big[\left\langle \nabla_A^i\phi_k \left|R^{2J-2}R^i\right| \nabla_B^j\nabla_B^j\phi_l\right\rangle - \left\langle \nabla_A^i\nabla_A^i\phi_k \left|R^{2J-2}R^j\right| \nabla_B^j\phi_l\right\rangle\\
&\qquad - (J+1)\left\langle \nabla_A^i\phi_k \left|R^{2J-2}\right| \nabla_B^i\phi_l\right\rangle - (J-1)\left\langle \nabla_A^i\phi_k \left|R^{2J-4}R^iR^j\right| \nabla_B^j\phi_l\right\rangle\Big]
\end{aligned}
$$

$$
\begin{aligned}
&\frac{4\,\pi}{2\,J+1}\sum_{M=-J}^{J}\left\langle p_A^2\psi_{k,\Pi_a}^{J,M}\middle|p_B^2\psi_{l,\Pi_b}^{J,M}\right\rangle \qquad (13)\\
&= \left\langle \nabla_A^i\nabla_A^i\phi_k \left|R^{2J}r_a^kr_b^k\right| \nabla_B^j\nabla_B^j\phi_l\right\rangle\\
&\quad - (J^2-3J-2)\left\langle \nabla_A^i\phi_k \left|R^{2J-2}\,r_a^kr_b^k\,\delta^{ij}\right| \nabla_B^j\phi_l\right\rangle\\
&\quad + (5J^2-7J-2)\left\langle \nabla_A^i\phi_k \left|R^{2J-4}r_a^kr_b^kR^iR^j\right| \nabla_B^j\phi_l\right\rangle\\
&\quad - (J^2+J+2)\left\langle \nabla_A^i\phi_k \left|R^{2J-2}(r_a^jr_b^i+r_a^ir_b^j)\right| \nabla_B^j\phi_l\right\rangle\\
&\quad + (J-1)\left\langle \nabla_A^i\phi_k \left|R^{2J-4}\left[(r_{bA}^2-r_{bB}^2)(R^jr_a^i+R^ir_a^j)+(r_{aA}^2-r_{aB}^2)(R^jr_b^i+R^ir_b^j)\right]\right| \nabla_B^j\phi_l\right\rangle\\
&\quad + (J+1)(2J-1)\left[\left\langle \nabla_A^i\phi_k \left|R^{2J-2}r_a^kr_b^k\right| \nabla_A^i\phi_l\right\rangle + \left\langle \nabla_B^j\phi_k \left|R^{2J-2}r_a^kr_b^k\right| \nabla_B^j\phi_l\right\rangle\right]\\
&\quad + \frac{1}{2}\left[\left\langle \nabla_A^i\phi_k \left|R^{2J-4}(r_{aA}^2-r_{aB}^2)(r_{bA}^2-r_{bB}^2)\right| \nabla_A^i\phi_l\right\rangle + \left\langle \nabla_B^j\phi_k \left|R^{2J-4}(r_{aA}^2-r_{aB}^2)(r_{bA}^2-r_{bB}^2)\right| \nabla_B^j\phi_l\right\rangle\right]\\
&\quad + 4J(J+1)\left\langle \phi_k \left|R^{2J-4}r_a^kr_b^k\right| \phi_l\right\rangle
\end{aligned}
$$

$$
\begin{aligned}
&\frac{4\,\pi}{2\,J+1}\sum_{M=-J}^{J}\left\langle p_A^2\psi_{k,\Pi}^{J,M}\middle|p_B^2\psi_{l,\Sigma}^{J,M}\right\rangle \qquad (14)\\
&= \sqrt{2J(J+1)}\Big\{2\,(J-1)\Big[\left\langle \rho^i\nabla_A^i\phi_k \left|R^{2J-3}\right| R^j\nabla_B^j\phi_\Sigma\right\rangle + \left\langle \rho^j\nabla_B^j\phi_k \left|R^{2J-3}\right| R^i\nabla_A^i\phi_\Sigma\right\rangle\\
&\qquad + \left\langle \phi_k \left|R^{2J-3}\right| \rho^i(\nabla_B^i-\nabla_A^i)\phi_\Sigma\right\rangle + \left\langle \phi_k \left|R^{2J-3}\nabla_R^i\rho^i\right| R^i(\nabla_B^i-\nabla_A^i)\phi_\Sigma\right\rangle\Big]\\
&\qquad + \left\langle \rho^j\nabla_B^j\phi_k \left|R^{2J-1}\right| \nabla_A^i\nabla_A^i\phi_\Sigma\right\rangle - \left\langle \rho^i\nabla_A^i\phi_k \left|R^{2J-1}\right| \nabla_B^j\nabla_B^j\phi_\Sigma\right\rangle\\
&\qquad - \left\langle \phi_k \left|R^{2J-1}\nabla_R^i\rho^i\right| (\nabla_B^j\nabla_B^j+\nabla_A^i\nabla_A^i)\phi_\Sigma\right\rangle\Big\}
\end{aligned}
$$

3 Table S1. Convergence of the intermediate expectation values composing the relativistic correction, Eq. (2), for rotational levels $J = 1 - 4$ of H_2

Calculations were performed (in a.u.) using the nonadiabatic James-Coolidge (naJC) wave function. The CODATA 2022 recommended proton-to-electron mass ratio 1836.152 673 426(32) was used. K is the size of the naJC basis set employed, governed by Ω – the largest shell enabled.

Ω	K	MV	D_{en}	$D_{\mathrm{ee}} \cdot 10^2$	$B_{\mathrm{ee}} \cdot 10^2$	$B_{\mathrm{en}} \cdot 10^3$	$B_{\mathrm{nn}} \cdot 10^6$	E_{rel}
				$J = 1$				
9	49042	−1.623 594 201 5	1.416 272 571 79	5.057 082 650 8	−4.622 134 449 5	−1.356 783 354 7	1.932 816 464 632	−0.204 326 998 3
10	73164	−1.623 593 926 6	1.416 272 566 45	5.057 082 791 8	−4.622 133 525 7	−1.356 783 306 5	1.932 816 463 872	−0.204 326 718 0
11	105840	−1.623 593 838 2	1.416 272 575 97	5.057 082 840 7	−4.622 133 207 1	−1.356 783 320 6	1.932 816 463 708	−0.204 326 616 4
12	149408	−1.623 593 808 5	1.416 272 574 32	5.057 082 847 2	−4.622 133 146 7	−1.356 783 313 0	1.932 816 463 659	−0.204 326 587 7
	∞	−1.623 593 78(3)	1.416 272 573(2)	5.057 082 85(1)	−4.622 133 08(6)	−1.356 783 31(1)	1.932 816 463 6(1)	−0.204 326 56(3)
				$J = 2$				
9	49042	−1.618 997 265 2	1.412 231 294 75	5.029 931 872 6	−4.607 224 429 7	−1.353 439 215 2	2.130 411 681 083	−0.203 890 204 8
10	73164	−1.618 997 017 7	1.412 231 289 05	5.029 931 985 0	−4.607 223 633 3	−1.353 439 168 2	2.130 411 680 205	−0.203 889 953 8
11	105840	−1.618 996 956 6	1.412 231 297 60	5.029 932 014 4	−4.607 223 409 4	−1.353 439 179 6	2.130 411 679 995	−0.203 889 881 7
12	149408	−1.618 996 920 4	1.412 231 296 22	5.029 932 024 2	−4.607 223 331 8	−1.353 439 172 8	2.130 411 679 968	−0.203 889 846 0
	∞	−1.618 996 88(3)	1.412 231 296(2)	5.029 932 03(1)	−4.607 223 25(8)	−1.353 439 17(1)	2.130 411 679 9(1)	−0.203 889 81(3)
				$J = 3$				
9	49042	−1.612 199 114 4	1.406 251 632 19	4.989 699 255 7	−4.585 065 552 4	−1.348 488 999 9	2.419 879 310 417	−0.203 247 214 3
10	73164	−1.612 198 874 9	1.406 251 626 59	4.989 699 363 6	−4.585 064 776 9	−1.348 488 953 2	2.419 879 309 497	−0.203 246 971 5
11	105840	−1.612 198 804 9	1.406 251 635 48	4.989 699 399 3	−4.585 064 520 8	−1.348 488 965 5	2.419 879 309 206	−0.203 246 889 7
12	149408	−1.612 198 768 7	1.406 251 634 17	4.989 699 409 2	−4.585 064 442 7	−1.348 488 958 9	2.419 879 309 263	−0.203 246 853 9
	∞	−1.612 198 74(3)	1.406 251 633(1)	4.989 699 42(1)	−4.585 064 36(8)	−1.348 488 96(1)	2.419 879 309 3(1)	−0.203 246 82(3)
				$J = 4$				
9	49042	−1.603 311 190 7	1.398 428 002 38	4.936 952 959 9	−4.555 894 666 4	−1.342 008 596 5	2.793 363 450 716	−0.202 411 820 6
10	73164	−1.603 310 944 6	1.398 427 997 48	4.936 953 072 7	−4.555 893 863 3	−1.342 008 552 1	2.793 363 449 755	−0.202 411 570 2
11	105840	−1.603 310 883 7	1.398 428 006 14	4.936 953 101 6	−4.555 893 639 8	−1.342 008 563 7	2.793 363 449 530	−0.202 411 498 1
12	149408	−1.603 310 850 8	1.398 428 004 73	4.936 953 109 7	−4.555 893 570 6	−1.342 008 556 8	2.793 363 449 492	−0.202 411 465 9
	∞	−1.603 310 82(3)	1.398 428 005(1)	4.936 953 12(1)	−4.555 893 50(7)	−1.342 008 56(1)	2.793 363 449 4(1)	−0.202 411 43(3)
